# First-Principles Investigation of Structural, Electronic, and Magnetic Properties of BiFeO_3_ and Bi_2_Fe_4_O_9_ Nanostructures

**DOI:** 10.3390/ijms26104671

**Published:** 2025-05-14

**Authors:** Ikbel Mallek-Zouari, Youness Kaddar, Wael Ben Taazayet, Omar Mounkachi, El-Kebir Hlil, Najeh Thabet Mliki, Amine El Moutaouakil

**Affiliations:** 1 Laboratory of Materials, Organization and Properties (LMOP), Faculty of Sciences of Tunis, University of Tunis El Manar, Tunis 2092, Tunisia; ikbel.mallek-zouari@fst.utm.tn (I.M.-Z.); waelbentaazayet@gmail.com (W.B.T.); najeh.mliki@fst.utm.tn (N.T.M.); 2 General Directorate of Technological Studies, Higher Institute of Technological Studies in Communications of Tunis (El Ghazala), Ariana 2083, Tunisia; 3 College of Computing, Mohammed VI Polytechnic University, Lot 660, Hay Moulay Rachid, Ben Guérir 43150, Morocco; younesskaddar@gmail.com (Y.K.); o.mounkachi@gmail.com (O.M.); 4 Laboratory of Condensed Matter and Interdisciplinary Sciences (LaMCScI), Faculty of Sciences, Mohammed V University in Rabat, Rabat 10112, Morocco; 5 Université Grenoble Alpes, CNRS, Grenoble INP, Institut Neel, 38000 Grenoble, France; el-kebir.hlil@neel.cnrs.fr; 6 Electrical and Communication Engineering, College of Engineering, UAE University, Al Ain P.O. Box 15551, United Arab Emirates

**Keywords:** bismuth ferrite, density functional theory (DFT), electronic properties, magnetic characterization

## Abstract

The structural, electronic, and magnetic properties of bismuth ferrite (BiFeO_3_) and Bi_2_Fe_4_O_9_ nanostructures were investigated using Density Functional Theory (DFT) within the Generalized Gradient Approximation (PBE-GGA) plus U approach. The PBE-GGA + U calculations predict band gaps of 2.4 eV for BiFeO_3_ and 2.3 eV for Bi_2_Fe_4_O_9_, closely aligning with experimental data. The analysis of partial and total density of states reveals strong hybridization between iron 3d and oxygen 2p states, with a significant contribution from Fe 3d orbitals in both structures. Additionally, nanostructure and crystal symmetry are crucial in influencing the magnetic properties of BiFeO_3_ and Bi_2_Fe_4_O_9_. Our calculations indicate that the antiferromagnetic phase is energetically more favorable than the ferromagnetic phase in both materials.

## 1. Introduction

Research on environmentally friendly energy sources has gained significant momentum in recent years. Solar-driven water splitting (WS) into H_2_ and oxygen (O_2_) using multiferroic materials as photocatalysts is considered a promising and innovative approach for hydrogen production and clean water purification.

Multiferroic materials exhibit interconnected electric, magnetic, and structural order parameters, leading to simultaneous ferromagnetic, ferroelectric, and ferroelastic states [1]. Bismuth ferrites such as BiFeO_3_ (BFO) and Bi_2_Fe_4_O_9_ are promising photocatalyst candidates. For BiFeO_3_, which exhibits an antiferromagnetic (AFM) spin arrangement, the Néel temperature (T_N_) is approximately 643 K, and the Curie temperature (Tc) is around 1100 K [2]. Meanwhile, Bi_2_Fe_4_O_9_ exhibits ferroelectric behavior with Curie and Néel temperatures of approximately 250 K and 265 K, respectively [3].

The unique multiferroic properties of bismuth ferrites have attracted significant interest for potential applications in innovative magnetoelectric and photovoltaic devices [4]. Recently, extensive efforts have been made to improve the efficiency of bismuth ferrite for applications such as wastewater degradation [5].

Numerous theoretical and experimental investigations have been conducted to enhance the electrical and magnetic properties of bismuth ferrites [6,7,8]. Petkov et al. [9] demonstrated that BiFeO_3_ nanoparticles retain a polar rhombohedral structure, exhibit ferromagnetism, and show enhanced magnetoelectric coupling. Using first-principles calculations, Campetella et al. [10] reported that suspended stoichiometric BiFeO_3_ flakes in the two-dimensional limit exhibit enhanced tetragonality and large dipole moments per unit surface. Additionally, density functional calculations, including GGA + U approximation, have been employed to study the influence of intrinsic vacancies on the electronic structure, magnetism, and dielectric functions of BiFeO_3_, revealing significant impacts on the total density of states and magnetic behavior [11]. The electronic structure of BiFeO_3_ in ferroelectric and paraelectric phases points out the presence of covalent bonding contribution, with a band gap of 1.198 eV and 0.280 eV, respectively [12].

In previous experimental work, we demonstrated that varying the annealing time results in distinct morphologies of BiFeO_3_ and Bi_2_Fe_4_O_9_. BiFeO_3_ nanoparticles with sizes below 10 nm and plate-like particles with dimensions of 100–200 nm (thickness of 30 nm) were successfully synthesized [13]. Studies have shown that morphology strongly influences the physical properties of these materials. Theoretical simulations have been employed to investigate electronic structures and magnetic properties. For instance, Sahoo et al. studied the magnetic behavior of Bi_2_Fe_4_O_9_ nanoparticles and found that coercivity, Neel temperature, and remanent magnetization depend strongly on particle shape, with sphere-like nanoparticles exhibiting a dominating antiferromagnetic component [14].

Despite extensive studies on BiFeO_3_ and Bi_2_Fe_4_O_9_, research on the relationship between morphology and theoretical properties remains limited.

Our previous work with Mössbauer spectroscopy reveals an asymmetry in the Fe^3+^-sextet spectrum for BiFeO_3_, indicating that the spin cycloid (bulk period of 62 nm) persists in nanoparticles significantly smaller than this threshold [15]. However, due to magnetic anisotropy induced by magnetoelastic and surface-confinement effects, the cycloid becomes unstable under a magnetic field of 0.2 T, transitioning into a homogenous antiferromagnetic state. More interestingly, increasing the external magnetic field up to 8 T results in multiple Mössbauer sextets attributed to a flexomagnetic effect due to strain gradients across the particle. The impact of the size of BFO micro-crystallites on the Mössbauer and magnetic properties has been examined and discussed in previous work [15]. Macroscopic magnetic measurements and in situ Mössbauer spectrometry were combined as a local probe of magnetization to investigate the magnetic response and stability of the synthesized BFO nanostructures. We demonstrated that the variation in the Mössbauer spectrum with temperature reflects the magnetic properties and arrangement of iron ions, and the transition to a magnetically split spectrum at 77 K indicates the initiation of magnetic ordering in the material. This study focused on the characterization of BiFeO_3_ nanoparticles and Bi_2_Fe_4_O_9_ synthesized via the hydrothermal method, enabling control over particle size, shape, and uniformity. Previous investigations involved X-ray diffraction (XRD), scanning electron microscopy (SEM), transmission electron microscopy (TEM), and UV–vis spectroscopy to characterize the obtained samples [13].

Although considerable experimental data on BFO ferrites exist, combined experimental and theoretical research investigations remain limited. The fundamental mechanisms governing the functional response, phase transformation, and magnetoelectric coupling in BFO have yet to be fully elucidated. In the present study, we employed Density Functional Theory (DFT) calculations based on the exchange correlation functional within the LDA + U approximation to explore structural phase transformations and magnetic and optical behavior.

The integration of experimental findings with Quantum ESPRESSO simulations offers a valuable approach to better understanding the surface stability, electronic properties, and magnetic behavior of bismuth ferrites. However, accurately capturing the complexities of these materials remains a challenge [16,17].

## 2. Results and Discussion

### 2.1. Structural Properties

**Figure 1:**
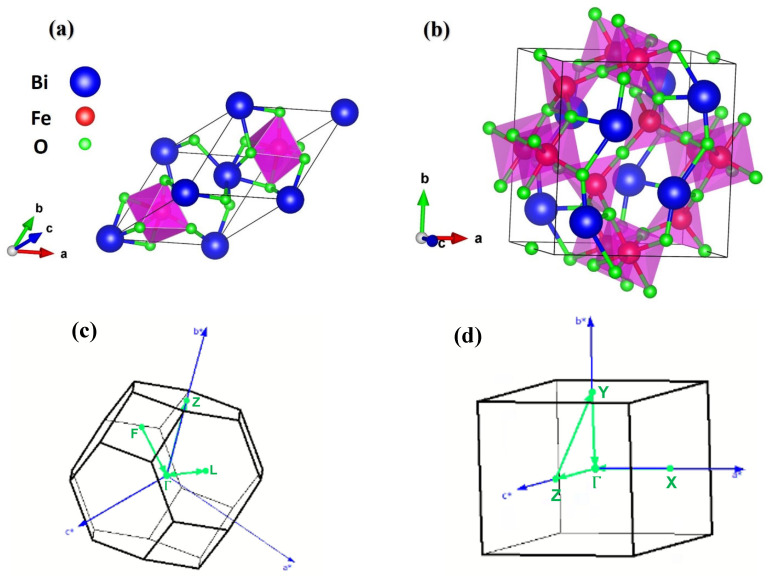
Crystal structures of (**a**) perovskite-type BiFeO_3_ in space group *R3c*, (**b**) mullite-type Bi_2_Fe_4_O_9_ in space group *Pbam*, and (**c**,**d**) Brillouin zones of the BiFeO_3_ and Bi_2_Fe_4_O_9_ structures, respectively, with the high symmetry points marked with green lines.

At ambient conditions, BiFeO_3_ crystallizes in a rhombohedral distorted perovskite structure (*R3c space group*), as illustrated in Figure 1a, whereas Bi_2_Fe_4_O_9_, adopts an orthorhombic structure (*Pbam space group*), as shown in Figure 1b. The optimized lattice parameters of these systems are presented in Table 1. Fe and O in the crystal structure, which is made up of a ${Fe}^{4+}O_{6}^{2-}$ octahedral and ${Fe}^{3+}O_{4}^{2-}$ tetrahedral. Fe, Bi, and O atoms have the following valence states: Fe (3p^6^3d^6^4s^2^), Bi (5d^10^6s^2^6p^3^) and O (2s^2^2p^4^). The band structures of BiFeO_3_ and Bi_2_Fe_4_O_9_ are calculated along high-symmetry directions (F–Γ–L–Γ–Z) and (X–Γ–Z–Y–Γ), respectively, in the Brillouin zones (refer to Figure 1c,d for notation).

To investigate the structural features of bismuth ferrite (Figure 1), we first optimize the crystalline structures of BiFeO_3_ and Bi_2_Fe_4_O_9_ (Table 1). The structural optimization was carried out using a magnetic structure to find the optimal lattice parameters that correspond to the system at an equilibrium state. As can be noted in Table 1, the estimated lattice parameters are close to the previous study [13,18,19]. In addition, our calculations conclude that the antiferromagnetic phase is more stable than the ferromagnetic one in both structures of BFO.

**Table 1:** Lattice parameters and bandgap energy E_g_ values of the bismuth ferrite system calculated using PBE-GGA + U approximation.

	This Work	Experimental Result [13]	Literature
**BiFeO_3_**			
**a = b = c (Å)**	5.6360(1)	5.5827(2)	5.59–5.69 [18,20,21,22]
α (°)	59.28	60	59–60 [20,21,22,23]
**E_g_ (eV)**	2.4	2.2	1–2.7 [20,21,23,24]
**Bi_2_Fe_4_O_9_**			
**a (Å)**	7.9740(1)	7.9431(4)	7.905–7.96 [19,25]
**b(Å)**	8.4240(1)	8.5276(2)	8.428–8.44 [19,25]
**c (Å)**	5.8670(1)	6.0291(2)	5.99–6.005 [19,25]
α (°)	90	90	90 [19,25]
**E_g_ (eV)**	2.3	2.34	1–2.4 [19,25,26]

### 2.2. Electronic Properties

The total and partial density of state (T/PDOS) and band structure of BiFeO_3_ and Bi_2_Fe_4_O_9_ were evaluated using PBE-GGA and PBE-GGA + U approximations.

**Figure 2:**
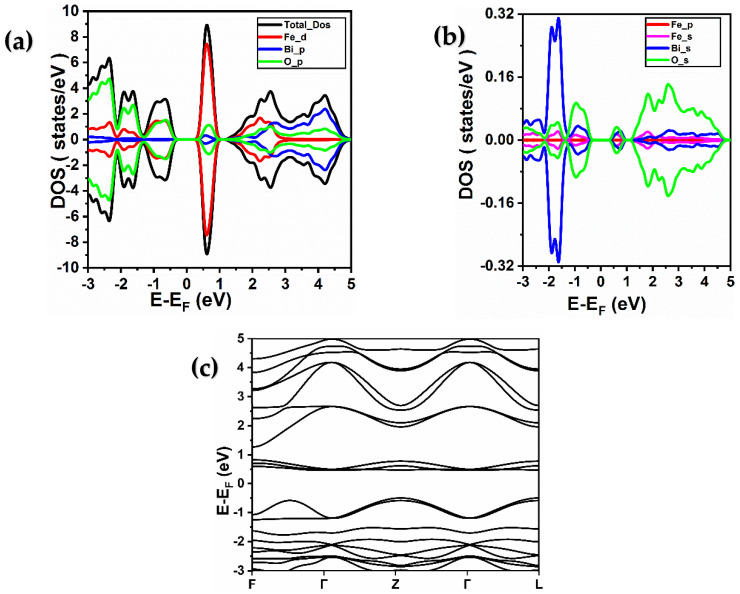
(**a**,**b**) Total and partial spin-resolved densities of states of BiFeO_3_ calculated using PBE-GGA approximation. (Fe (d), Fe (p), and Fe (s) denote the 3d, 3p, and 4s orbitals of iron, respectively, while Bi (p) and Bi (s) represent the 6p and 6s orbitals of bismuth, respectively, and O (p) and O (s) signify the 2p and 2s orbitals of oxygen, respectively). (**c**) Band structure of BiFeO_3_ material obtained using PBE-GGA approximation.

**Figure 3:**
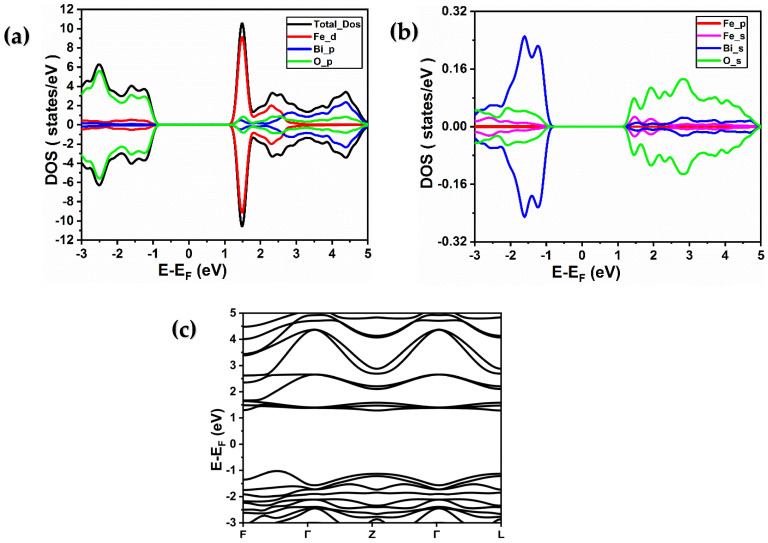
(**a**,**b**) Total and partial spin-resolved densities of states of BiFeO_3_ calculated using PBE-GGA + U (4 eV) approximation. (Fe (d), Fe (p), and Fe (s) denote the 3d, 3p, and 4s orbitals of iron, respectively, while Bi (p) and Bi (s) represent the 6p and 6s orbitals of bismuth, respectively, and O (p) and O (s) signify the 2p and 2s orbitals of oxygen, respectively). (**c**) Band structure of BiFeO_3_ material obtained using PBE-GGA + U (4 eV) approximation.

For BiFeO_3_, the density of states is shown in Figure 2a and Figure 3a. As observed, the 3d states of iron with red color are responsible for most of the density of the state. The BiFeO_3_ material distortions allow the ${Fe}^{4+}$ to move along the octahedron axis. Furthermore, as can be seen in Figure 2a and Figure 3a, the density of states resulting from the 6p and 2p states of bismuth and oxygen, respectively, exhibit a significant value. Furthermore, a small density of states value is seen in the (4s) and (6s and 6p) and (2s) states of iron, bismuth, and oxygen, respectively, according to the density of states in Figure 2b and Figure 3b. The Fe-O band is determined by the chemical environment and the hybridization of the iron 3d and oxygen 2p states. The DOS of the Fe element takes place near the Fermi level, with the 3d orbital accounting for a considerable fraction. The 3d-Fe states exhibit polarization, pointing to a semiconductor behavior for BiFeO_3_. Shen et al. [12] reported that the analysis of the electronic structures of BiFeO_3_ in ferroelectric and paraelectric phases indicates that the O-2p electrons hybridize with Bi-6p and Fe-3d electrons, which suggests the presence of covalent bonding contribution in BiFeO_3_. Moreover, the calculated value of the band gap using PBE-GGA approximation, as shown in Figure 2c, is about 1 eV. However, experimental data [13] indicate a band gap value of about 2.4 eV. As a result, these orbitals 3d of iron, using GGA + U correction with the values for U = 4 eV, demonstrate that 3d is at 1.5 eV with a band gap of 2.4 eV (as shown in Figure 3c and Table 1), which is consistent with that reported in another work [20,21,23,27].

**Figure 4:**
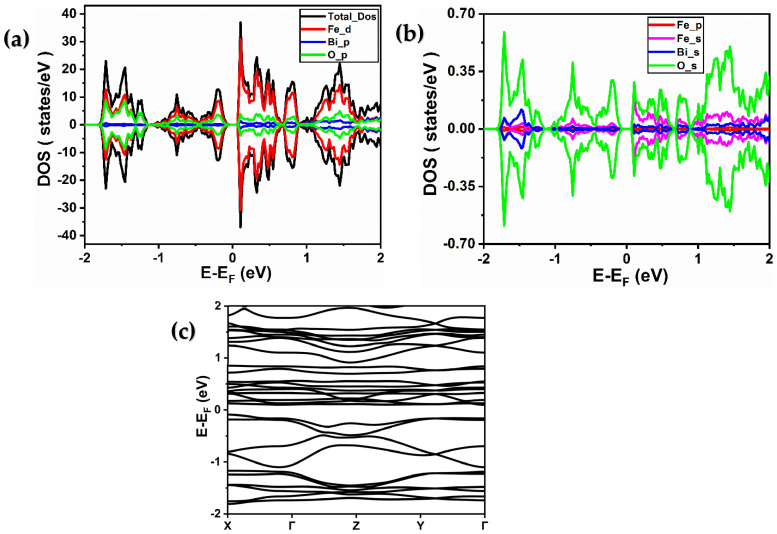
(**a**,**b**) Total and partial spin-resolved densities of states of Bi_2_Fe_4_O_9_ calculated using PBE-GGA approximation. (Fe (d), Fe (p), and Fe (s) denote the 3d, 3p, and 4s orbitals of iron, respectively, while Bi (p) and Bi (s) represent the 6p and 6s orbitals of bismuth, respectively, and O (p) and O (s) signify the 2p and 2s orbitals of oxygen, respectively). (**c**) Band structure of BiFeO_3_ material obtained using PBE-GGA approximation.

**Figure 5:**
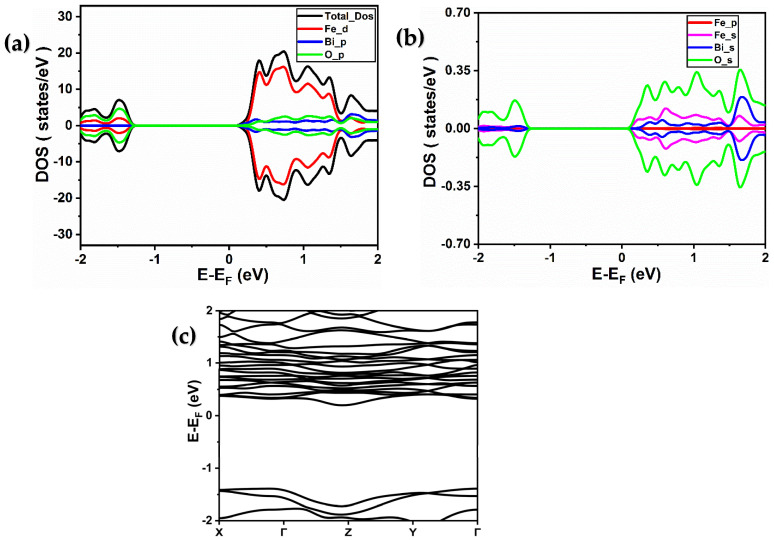
(**a**,**b**) Total and partial spin-resolved densities of states of Bi_2_Fe_4_O_9_ calculated using PBE-GGA + U (4 eV) approximation. (Fe (d), Fe (p), and Fe (s) denote the 3d, 3p, and 4s orbitals of iron, respectively, while Bi (p) and Bi (s) represent the 6p and 6s orbitals of bismuth, respectively, and O (p) and O (s) signify the 2p and 2s orbitals of oxygen, respectively). (**c**) Band structure of BiFeO_3_ material obtained using PBE-GGA + U (4 eV) approximation.

For the second structure, Bi_2_Fe_4_O_9_ presented in Figure 1b, the distortion in the Bi_2_Fe_4_O_9_ material allows for the movement of ${Fe}^{3+}$ along the tetrahedral axis and ${Fe}^{4+}$ along the octahedron axis. The electronic structure of Bi_2_Fe_4_O_9_ can be obtained from its total and partial density of states (T/PDOS) and band structure, as shown in Figure 4 and Figure 5 for GGA and GGA + U, respectively. Also, the Bi_2_Fe_4_O_9_ exhibits a semiconductor character. The density of states shows the hybridization of the iron 3d and oxygen 2p states with a large contribution due to the 3d orbitals of iron atoms. Figure 4c and Figure 5c depict the computed band structure in the highly symmetric directions in the first Brillouin zone. Bi_2_Fe_4_O_9_ is a semiconductor with a band gap value at the Γ point around 1 eV and 2.3 eV using GGA and GGA + U, respectively [26]. So, the calculations confirm that both materials exhibit semiconductor behavior, with Fe 3d and O 2p hybridization significantly influencing electronic structure.

### 2.3. Magnetic Structure and Magnetic Anisotropy

Magnetic properties of bismuth ferrite are primarily due to the polarization of Fe ions. The values of the induced magnetic moments at atomic sites depend on their local environments. This is clearly shown in our case, in which an ${Fe}^{4+}O_{6}^{2-}$ octahedral formed between oxygen and iron ions in the BiFeO_3_ structure gives a magnetic moment value of Fe atoms of about 3.81 ${µ}_{B}$, as shown in Table 2. However, in the Bi_2_Fe_4_O_9_ structure, the oxygen and iron ions formed an ${Fe}^{4+}O_{6}^{2-}$ octahedral and an ${Fe}^{3+}O_{4}^{2-}$ tetrahedral, which give a magnetic moment values of Fe atoms of around 3.67 ${µ}_{B}$ and 3.78 ${µ}_{B}$, respectively.

In agreement with experimental data, we proved in previous work, for Bi_2_Fe_4_O_9_, the data were fitted with two paramagnetic components, unambiguously corresponding to ferric species in tetrahedral and octahedral units [30].

Magnetic anisotropy in magnetic materials results from spin–orbit coupling. This permits orbitals to align themselves with one or more crystallographic axes, resulting in an easy magnetization direction. The magnetic anisotropy energy is typically determined by the symmetry of the crystalline structure, spin–orbit effects, and exchange interactions between magnetic moments. In fact, magnetic anisotropy is caused by the combined contributions of all these processes. We calculate the magnetic anisotropy energy for each direction, and the lowest absolute values correspond to the a-axis for BiFeO_3_. The results reveal that the a-axis corresponds to an easy magnetization direction for BiFeO_3_ crystal; the magnetic anisotropy value is (∆=−32 meV), in agreement with the experimental data [31]. Indeed, we demonstrated in previous work that as a consequence of a flexomagnetic effect arising from strain gradients due to a continuous variation in the coupling between magnetization and the structural distortion from the surface to the particle core [15].

This direction is favored due to the strong spin–orbit coupling and the Dzyaloshinskii–Moriya interaction in the material, which influences its multiferroic properties, including its magnetic behavior. In addition to strain effects, magnetic interactions are also altered at the surface due to reduced symmetry, giving rise to surface magnetic anisotropy. Recent calculations [32] have demonstrated that this anisotropy depends on the orientation of the surface normal relative to the cycloid propagation vector, leading to distinct “proximity” and “edge” effects. However, for Bi_2_Fe_4_O_9_ material, the results reveal that the b-axis corresponds to an easy magnetization direction with the magnetic anisotropy value (∆=−7.9045 meV). As the particle size decreases, the surface effect and anisotropy increase, eventually leading the particles to enter the multi-domain region. We illustrated in a previous experimental study that the increase in magnetization can be attributed to the increase in the surface-to-volume ratio resulting from the reduction in particle size. This increase leads to a total dipole moment induced by the canted, irregular, and non-collinear spin order within the material [30].

The calculated density of states shows significant hybridization between Fe-3d states and O-2p states along with minor overlap between Bi-6p and O-2p states. The magnetic properties of Bi_2_Fe_4_O_9_ are primarily due to Fe^3+^ ions, each of which has a non-integer magnetic moment of approximately 3.9 ${µ}_{B}$. The values of the induced magnetic moments at other atomic sites depend on their local environments [26]. Verseils et al. claimed that the crystal structure of Bi_2_Fe_4_O_9_ influences its magnetic properties through spin-lattice coupling, as evidenced by anomalous softening of phonon modes in the antiferromagnetic phase [33]. The magnetic properties exhibit a combination of ferromagnetic and antiferromagnetic, with the potential for unusual magnetic phase transitions [34].

## 3. Materials and Methods

First-principles calculations based on a DFT method were employed to investigate the ground state nature of BiFeO_3_ and Bi_2_Fe_4_O_9_. The calculations were performed using the plane-wave (PW) pseudopotential method implemented in the Quantum ESPRESSO code [35]. Exchange and correlation effects were accounted for using a Perdew–Burke–Ernzerhof (PBE)-Generalized Gradient Approximation (PBE-GGA) [36]. To correct the band gap values, the GGA + U approach, with U = 4 eV, was used. This correction adds an on-site Coulomb interaction term (GGA + U) to describe localized electron interactions more accurately [37]. The plane-wave cutoff energy was set to 70 Ry; it was selected after careful convergence testing to ensure a good balance between computational efficiency and the accuracy of total energy, forces, and electronic structure, and a Monkhorst–Pack k-point grid of 10 × 10 × 10 was used to sample the Brillouin zones. The convergence criterion was set at $10^{-5}$ eV.

## 4. Conclusions

This study highlights the strong correlation between nanostructure, crystal symmetry, and magnetic properties of BiFeO_3_ and Bi_2_Fe_4_O_9_. The calculations were performed at two levels of theory, namely DFT and DFT + U. The exchange-correlation (xc) functionals PBE and PBE + U with GGA pseudopotentials have been tested to compute diverse properties of BiFeO_3_ and Bi_2_Fe_4_O_9_. While all the exchange-correlation functionals accurately capture the structural properties, they fall short in accurately predicting their band structure. The introduction of the DFT + U method improves the calculated band structure. Specifically, PBE-GGA + U calculations predict a bandgap of 2.4 eV for BiFeO_3_ and 2.3 eV for Bi_2_Fe_4_O_9_, in accordance with our experimental results [20,21,23]. Nanoscale morphology and crystal structure strongly influence the magnetic properties of BiFeO_3_ and Bi_2_Fe_4_O_9_ related compounds. The results revealed that the a-axis corresponds to an easy magnetization direction for BiFeO_3_ crystal, and the b-axis corresponds to an easy magnetization direction for Bi_2_Fe_4_O_9_. The calculations can be used to infer that the nanostructuring effects and crystal structure strongly influence the magnetic properties of BiFeO_3_ and Bi_2_Fe_4_O_9_. It is important to report that the results of different magnetic configurations proved the hypothesis that nanostructuring significantly impacts the magnetic behavior and structural properties of BiFeO_3_ and Bi_2_Fe_4_O_9_. It shows that the antiferromagnetic phase is more stable than the ferromagnetic one in both materials. Magnetic anisotropy energy calculations indicate that BiFeO_3_ favors magnetization along the a-axis, whereas Bi_2_Fe_4_O_9_ prefers the b-axis.

The findings provide insights into the fundamental mechanisms governing multiferroic behavior, opening new possibilities for applications in electronic and magnetoelectric devices.

## Figures and Tables

**Table 2:** Magnetic moments of iron atoms of the bismuth ferrite system calculated using PBE-GGA + U approximation.

	This Work	Literature
**BiFeO_3_**		
**Magnetic moments of Fe (** ${µ}_{B}$ **)** **in an ** ${Fe}^{4+}O_{6}^{2-}$ ** octahedral**	3.8168	3.75 [28]
**Bi_2_Fe_4_O_9_**		
**Magnetic moments of Fe** **in an ** ${Fe}^{4+}O_{6}^{2-}$ ** octahedral (** ${µ}_{B}$ **)**	3.6777	3.9 [26]
**Magnetic moments of Fe** **in an ** ${Fe}^{3+}O_{4}^{2-}$ ** Tetrahedral (** ${µ}_{B}$ **)**	3.7854	4.95 [29]

## Data Availability

The datasets used and/or analyzed during the current study are available upon reasonable request.
